# Brain MRI White Matter Hyperintensity and Microbleed Associations in Trauma Patients With CT-Detected Intracranial Macrohemorrhage: A Retrospective Study

**DOI:** 10.7759/cureus.105775

**Published:** 2026-03-24

**Authors:** C. Michael Dunham, Gregory S Huang, Kene T Ugokwe, Brian P Brocker, Renee Merrell

**Affiliations:** 1 Trauma, Critical Care, and General Surgery, St. Elizabeth Youngstown Hospital, Youngstown, USA; 2 Neurosurgery, St. Elizabeth Youngstown Hospital, Youngstown, USA; 3 Trauma, St. Elizabeth Youngstown Hospital, Youngstown, USA

**Keywords:** adverse outcomes, brain axonal injury, brain magnetic resonance imaging, glasgow coma scale, trauma centers, traumatic intracranial hemorrhage, white matter hyperintensity, white matter microbleed

## Abstract

Objective

The present study aimed to compare white matter hyperintensity (WMH) and white matter microbleed (WMM) proportions in trauma patients with intracranial hemorrhage (ICH) to published participants without ICH. When WMH or WMM proportions were associated with head trauma, these proportions were assessed to determine their associations with multiple undesirable outcomes.

Methods

The study population had ICH on CT, an admission Glasgow Coma Scale (GCS) score of 3-15, and blunt trauma. MRI was reviewed to determine the presence of WMH and WMM. The prevalence of non-trauma WMH and WMM by age was collated from the literature. The CT ICH mass effect (ME) scores reflect the presence or absence of lateral ventricular compression, basal cistern compression, and midline shift (0-3). The CT ICH Abbreviated Injury Scale (AIS) scores were obtained from the trauma registry (1-5). The total ICH score was determined by calculating AIS + ME + WMM (presence = 1; absence = 0) (range 1-9). An adverse outcome included hospital deaths, intensive care stay ≥2 days, or hospital stay >5 days. Multivariate analyses were used to determine independent associations.

Results

Of 1,545 patients, MRI was performed in 173 (mean age, 64.7 years). The WMH proportion was lower in patients with ICH (117/173; 67.6%) than in non-ICH age-matched counterparts (3,385/4,073; 83.1%; p < 0.0001). Mean ages with and without WMH were 72.4 and 48.6 years, respectively (p < 0.0001). The WMM proportion was greater in patients with ICH (96/173; 55.5%) than in non-ICH age-matched counterparts (473/3,216; 14.7%; p < 0.0001). Macrobleed on CT (ME + AIS scores) had univariate associations (p < 0.02 to < 0.001) with hospital deaths, intensive care and hospital lengths of stay, an adverse outcome, and failure to follow commands at discharge. Hospital mortality had an independent association with total ICH score (GCS 3-12, p > 0.1000; ME + AIS score, p > 0.1000; total ICH score, p = 0.0151). An ICU stay of ≥2 days was independently associated with total ICH score (GCS 3-12, p = 0.0004; ME + AIS score, p > 0.1000; total ICH score, p = 0.0004). Similarly, a hospital stay exceeding five days was significantly associated with total ICH score (GCS 3-12, p = 0.0004; ME + AIS score, p > 0.1000; total ICH score, p = 0.0071). An adverse outcome also showed an independent association with total ICH score (GCS 3-12, p = 0.0031; ME + AIS score, p > 0.1000; total ICH score, p = 0.0046). Additionally, failure to follow commands at three months was independently associated with total ICH score (GCS 3-12, p = 0.8283; ME + AIS score, p = 0.0948; total ICH score, p = 0.0443). The ME + AIS score did not show significant independent associations with these outcomes (p > 0.1000).

Conclusions

WMMs were indicative of traumatic axonal brain injury, whereas WMHs were associated with advancing age. Macrobleed on CT had univariate associations with multiple undesirable outcomes. The combination of macrobleeds identified on CT (ME + AIS) and microbleeds detected on MRI (WMM) showed independent associations with poor outcomes. However, CT-detected macrobleeds without microbleed status did not demonstrate an independent association. Findings from this study underscore the need for further investigation into the quantification of CT macrobleeds and MRI microbleeds and their correlation with both in-hospital and long-term clinical outcomes.

## Introduction

Some investigators have included the presence of a brain white matter hyperintensity (WMH) as a manifestation of post-traumatic brain axonal injury [1-4], whereas others have not [5-8]. All of these investigators acknowledge the presence of brain white matter microbleeds (WMMs) as an indication of post-traumatic brain axonal injury [1-8]. Complicating this controversy, literature indicates that both WMH and WMM have also been associated with the aging process. Some investigators have shown that WMH proportions increase with advancing age [9,10], while others have demonstrated a similar finding for WMM proportions [11,12].

Of relevance to the current study, multiple investigators have reported that head Abbreviated Injury Scale (AIS) scores are associated with hospital mortality in trauma patients [13-15]. Researchers have also shown that head AIS scores are higher in trauma patients with increased ICU length of stay [14] and 12-month unfavorable Glasgow Outcome Scale (GOS) scores [16].

Observations that traumatic intracranial mass effect (ME) adversely affects patient outcomes are also germane to the present study. The Marshall categorical system and the Rotterdam CT score, which contain elements for delineating MEs, have been extensively investigated in trauma patients with head injury. Several studies have shown that these early CT score findings are associated with in-hospital mortality [17-21]. Numerous research studies have also demonstrated that these CT scores are associated with post-hospital unfavorable GOS scores [21-25]. Furthermore, an investigation of patients with traumatic brain injury reported that the ME score was a better indicator of the need for surgical decompression than the GCS score [26].

In the current study, the early post-injury Glasgow Coma Scale (GCS) score, a measure of brain function, is a fundamental indicator of traumatic brain injury. Early post-injury GCS has been associated with both in-hospital mortality [22,27,28] and post-hospital discharge GOS scores [22-24,27-30]. Previous investigations regarding post-traumatic brain axonal injury have typically excluded patients with an admission GCS of 13-15 [1,6-8,31]. To our knowledge, the only study on post-traumatic brain axonal injury that included patients with the full GCS spectrum (3-15) examined only patients with a favorable post-discharge outcome [32].

Previous publications have defined six undesirable trauma outcomes: hospital mortality, intensive care stay ≥2 days, hospital stay >5 days, adverse outcome (any of the previous three outcomes), failure to follow commands at hospital discharge, and failure to follow commands at three months [26,33-35]. The current study assessed trauma center patients who had an intracranial hemorrhage (ICH), a blunt trauma mechanism, and a brain MRI within 60 days of injury.

The first objective of the study was to compare the proportions of WMH and WMM observed on MRI in patients with head trauma to published proportions in age-matched individuals without head injury. The second aim was to evaluate associations between increased WMH and/or WMM on MRI and the six predefined post-injury adverse outcomes in patients with ICH. The third objective involved comparing GCS scores of 3-12, the combination of head AIS + ME, and total ICH scores with the same six undesirable outcomes. Finally, multivariate analyses were conducted to identify independent associations between GCS 3-12, ICH AIS + ME, and total ICH scores with the six undesirable outcomes.

## Materials and methods

Ethics statement

This study was reviewed by the local institutional review board (IRB) and determined to qualify for exemption according to Exempt Category 4 (Bon Secours Mercy Health, approval 2025-TRAUMA-Dunham). The need for informed consent was waived due to minimal risk and the retrospective nature of the investigation. All medical records were de-identified.

Study design and population

In the current retrospective study, the parent data source consisted of adult blunt trauma patients with ICH admitted to a Level I trauma center in Northeast Ohio. The time periods included January 21 to July 21 for each year, 2018, 2019, and 2020; and January 1 to December 31 for each year, 2021, 2022, 2023, and 2024.

Inclusion and exclusion criteria

Data from the trauma registry were used to identify the parent cohort of trauma center admissions aged ≥18 years with blunt trauma and ICH. The electronic medical record (EMR) of patients with a hospital stay of ≥3 days was reviewed to determine if an MRI had been performed within 60 days of ICH injury. The current study included only those who had undergone an MRI. Patients were excluded if they had no MRI, were aged <18 years, had a non-blunt trauma mechanism, or had no ICH.

Data collection

The parent group with a hospital length of stay of ≥3 days underwent an EMR review to determine if a brain MRI had been performed within 60 days following ICH injury. Blunt trauma patients with ICH who had a post-injury MRI comprised the principal group of focus for the current study.

Trauma patient WMH and WMM proportions and comparisons with non-ICH published data

Each MRI trauma patient’s T2-weighted fluid-attenuated inversion recovery (FLAIR) images were reviewed for the presence of any WMH (yes = 1; no = 0), and T2-gradient echo or susceptibility-weighted images were reviewed for the presence of any WMM (yes = 1; no = 0). All MRI images were reviewed and interpreted by the first author, a trauma surgeon, board-certified intensivist, and general surgeon with over 30 years of experience. During each MRI review, no patient’s age, GCS score, outcomes, or brain CT images were assessed to avoid influencing MRI interpretation.

All study patient brain MRI images were generated using a General Electric Medical Systems Signa HDxt scanner with a 1.5 Tesla magnetic field strength (GE HealthCare, Chicago, Illinois). All brain MRI images included an axial FLAIR sequence and either an axial gradient echo or an axial susceptibility-weighted sequence. All brain CT images were generated using a General Electric Medical Systems or Siemens Somatom scanner (Siemens AG, Munich, Germany) with 128 detector rows, employing a helical imaging technique with a rotation time of 0.23-0.35 seconds.

To compare trauma patient WMH and WMM proportions with age-associated non-trauma patient proportions, partial, non-comprehensive searches were conducted. WMH age-associated prevalence publications were identified using the Google Chrome search term “white matter hyperintensity prevalence”. Studies were included if cited in PubMed and if patients with underlying brain disease were excluded [9,10,36-43]. WMM age-associated prevalence publications were identified using the Google Chrome search term “white matter microbleed prevalence” and included if cited in PubMed and if patients with underlying brain disease were excluded [11,12,44-49]. Reference tables were constructed from these publications, depicting WMH and WMM proportions according to participant age.

One study objective was to compare ICH trauma patient WMH and WMM proportions with age-matched non-head-injured patient proportions. The mean age of the ICH trauma patients was used to select age-related data from the WMH and WMM reference tables. If the trauma and non-trauma WMH or WMM proportions were similar, this would suggest that findings were age-related. If ICH trauma WMH or WMM proportions exceeded those of the non-head-injured reference group, this would suggest that the trauma patient proportions were related, at least in part, to head injury.

Outcomes

Study patient outcomes included hospital mortality, ICU stay ≥2 days, hospital stay >5 days, any adverse outcome, not following commands at hospital discharge, and not following commands at three months. Data on hospital mortality, ICU stay ≥2 days, and hospital stay >5 days were obtained from the trauma registry. Adverse outcome was classified as “yes” if the patient had (1) hospital mortality; (2) ICU stay ≥2 days; or (3) hospital stay >5 days [34,35].

Patients were classified as not following commands at hospital discharge if (1) they died during the trauma center stay (trauma registry) or (2) EMR review of hospital survivors demonstrated that they did not follow commands at discharge [26,33]. Patients were classified as not following commands at three months if (1) they died during the trauma center stay (trauma registry); (2) a hospital survivor died within three months of discharge (EMR review); or (3) they were alive at three months but did not follow commands (EMR review) [26,33].

Risk conditions

Study patient risk conditions included the presence of WMH, the presence of WMM, ICH AIS + ICH ME score, total ICH score, GCS, and GCS deficit + total ICH score. WMH and WMM assessments were performed as previously described. Head AIS scores, as described in an open-access article [14], were obtained from the trauma registry. Because all study patients had ICH, the head AIS score is referred to as the ICH AIS score. A review of patient ICH AIS codes was performed to determine the proportion based on traits of ICH observed on admission CT. The ICH ME score was computed from the admission brain CT scan based on lateral ventricular compression (1 = yes), midline shift (1 = yes), and basal cistern compression (1 = yes), with a range of 0-3 [26,33]. The ICH AIS + ICH ME score was computed as the sum of the two components (range 2-8). The total ICH score was computed as the sum of the ICH AIS score (1-5), ICH ME score (0-3), and WMM score (0-1), with a range of 1-9. GCS (range 3-15) was obtained from the trauma registry and used to calculate a GCS deficit (15 minus GCS; range 0-12) [26]. The GCS deficit + total ICH score was computed as the sum of these two values (range 1-21).

Statistical analysis

All analyses were performed on the 173 ICH patients who had an MRI. All analyses included these 173 patients, except for one patient in whom the following commands at three months could not be classified. Published WMH and WMM proportions at varying ages were collated from the literature. Each ICH MRI GCS group (3-15, 13-15, or 3-12) had a specific mean age. Age-matched non-ICH literature WMH and WMM proportions were used for comparison with each ICH MRI GCS group. When multiple citations were available for a proportion, a combined proportion was computed. Random-effects combined proportions were used when inter-study heterogeneity existed; fixed-effects combined proportions were used when heterogeneity did not exist.

Continuous data are presented as mean ± standard deviation, proportional data as count (N) and percent, and ordinal data as median and interquartile range. T-tests and Cohen’s d values were computed to assess differences between two groups of continuous data (age). For dichotomous proportional data in 2 × 2 contingency tables (WMH, WMM, GCS 3-12, GCS 13-15, AIS + ME ≥5, total ICH score ≥6), the two-tailed Fisher’s exact test was used when any expected cell frequency was <5; the chi-square test was used. ORs and risk ratios (RR) were computed to quantify proportional differences.

The Wilcoxon rank sum test was used to compare medians between two independent groups (AIS + ME, total ICH score, GCS deficit, GCS deficit + total ICH score). The Wilcoxon signed-rank test was used for paired comparisons of GCS deficit and GCS deficit + total ICH score in patients not following commands at hospital discharge. Spearman correlation coefficients were computed for the relationship between ICH AIS and ICH ME scores. Cut points for AIS + ME and total ICH score were assessed using receiver operating characteristic (ROC) curve analyses. Multivariate logistic regression was used to assess simultaneous associations of independent variables with dichotomous dependent variables, including mortality, ICU stay ≥2 days, hospital stay >5 days, adverse outcome, no hospital discharge commands, and no commands at three months. Data were entered into Excel 2010 (Microsoft Corporation, Redmond, WA, USA) and imported into SAS System for Windows (release 9.2, SAS Institute Inc., Cary, NC, USA). ROC curve analyses were performed using MedCalc® Statistical Software (version 22.016; MedCalc Software Ltd., Ostend, Belgium). The significance level was set at p < 0.05.

## Results

The 2018-2024 database included 1,545 trauma center patients admitted with ICH and blunt trauma. EMR review of the 1,054 patients with hospital stays ≥3 days showed that a postinjury MRI had been performed in 173/1,054 (16.4%) patients. The performance of an MRI in those with a hospital stay of ≥3 days had no association with AIS (p = 0.0734) or GCS (p = 0.1855). However, those with MRI were slightly younger (64.7 vs. 68.1 years; p = 0.0391) and had a slightly higher proportion with an ICU length of stay ≥2 days (19.4% vs. 13.7%; p = 0.0129) than those without MRI. Common indications for MRI listed in the EMR included persistent coma, motor deficits (unsteady gait, focal facial weakness, and hemiparesis), pre-ICH confusion, new-onset post-ICH confusion, neurologic deterioration, and a brain mass on CT.

ICH patient associations with outcomes

Table 1 presents the univariate associations of GCS 3-12 and WMM proportions with outcomes. GCS 3-12 proportions were greater in patients with hospital mortality, increased ICU stay, increased hospital stay, adverse outcomes, and not following commands at hospital discharge compared with their counterparts. WMM proportions were greater in patients with increased ICU stay, increased hospital stay, not following commands at hospital discharge, and not following commands at three months compared with their counterparts.

**Table 1:** Univariate associations of GCS 3-12 and WMMs with outcomes GCS, Glasgow Coma Scale score; WMM, white matter microbleed

Outcome	No.	%	GCS 3-12 no. (%)	Fisher’s exact p-value	χ² p-value	χ²	WMM no. (%)	χ² p-value	χ²
Lived	162	93.6	37 (22.8)	-	-	-	87 (53.7)	-	-
Died	11	6.4	6 (54.6)	0.0185	-	-	9 (81.8)	0.0694	3.3
Intensive care stay <2 days	76	43.9	1 (1.3)	-	-	-	35 (46.1)	-	-
Intensive care stay ≥2 days	97	56.1	42 (43.3)	-	<0.0001	40.2	61 (62.9)	0.027	4.9
Hospital stay ≤5 days	73	42.2	4 (5.5)	-	-	-	34 (46.6)	-	-
Hospital stay >5 days	100	57.8	39 (39.0)	-	<0.0001	25.4	62 (62.0)	0.0438	4.1
Adverse outcome - no	57	32.9	1 (1.8)	-	-	-	26 (45.6)	-	-
Adverse outcome - yes	116	67.1	42 (36.2)	-	<0.0001	24.3	70 (60.3)	0.0669	3.4
Discharge commands	152	87.9	27 (17.8)	-	-	-	79 (52.0)	-	-
No discharge commands	21	12.1	16 (76.2)	-	<0.0001	33.7	17 (81.0)	0.0123	6.3
Three-month commands	142	82.6	34 (23.9)	-	-	-	74 (52.1)	-	-
No three-month commands	30	17.4	9 (30.0)	-	0.4864	1	22 (73.3)	0.0335	4.5

The proportions of the highest ICH AIS scores were as follows: 2, 27.8%; 3, 17.3%; 4, 37.0%; and 5, 17.9%, with a median of 4 and an interquartile range of 2-4. A review was undertaken to assess each AIS code used to determine the head AIS score for the MRI patients. Of the 173 patients, the review showed that the highest head AIS codes were based on CT traits of the intracranial macrohemorrhage in 162 (93.6%).

Table 2 presents the univariate differences between ICH ME + ICH AIS scores and total ICH scores for the six undesirable outcomes. Total ICH scores were greater than ICH ME + ICH AIS scores for all six undesirable outcomes. ICH AIS and ICH ME scores had a significant association (p < 0.0001); however, their correlation was moderate (Spearman r = 0.39).

**Table 2:** Univariate differences between ICH ME + ICH AIS scores and total ICH scores with undesirable outcomes ICH macrobleed = ICH AIS + ICH ME; total ICH = ICH macrobleed + ICH microbleed AIS, Abbreviated Injury Scale; ICH, intracranial hemorrhage; ME, mass effect

Outcome	No.	%	AIS + ME median (IQR)	Total ICH median (IQR)	Paired Wilcoxon p-value
Died	11	6.4	5 (3-7)	6 (3-8)	0.0027
Intensive care stay ≥2 days	97	56.1	4 (3-5)	5 (4-6)	<0.0001
Hospital stay >5 days	100	57.8	4 (3-5)	5 (4-6)	<0.0001
Adverse outcome - yes	116	67.1	4 (3-5)	5 (3.5-6)	<0.0001
No discharge commands	21	12.1	5 (4-6)	6 (5-7)	<0.0001
No three-month commands	30	17.4	4 (3-5)	5 (3-6)	<0.0001

Table 3 displays the OR and RR for each of the six undesirable outcomes in relation to ICH ME + ICH AIS ≥5 and total ICH ≥6. The ICH ME + ICH AIS cut point (≥5) and the total ICH cut point (≥6) were determined using ROC curve analyses (p < 0.0001). Of the six undesirable outcomes, five had increased proportions of ICH ME + ICH AIS ≥5, whereas all six had increased proportions of total ICH ≥6. For each undesirable outcome, the OR and RR were higher with total ICH ≥6 than with ICH ME + ICH AIS ≥5.

**Table 3:** Univariate associations of ICH ME + ICH AIS scores and total ICH scores with outcomes ICH macrobleed = ICH AIS + ICH ME; total ICH = ICH macrobleed + ICH microbleed AIS, Abbreviated Injury Scale; ICH, intracranial hemorrhage; ME, mass effect; RR, risk ratio

Outcomes	No.	%	AIS + ME ≥5 no. (%)	Fisher’s exact p-value	χ² p-value	χ²	OR	RR	Total ICH ≥6 no. (%)	Fisher’s exact p-value	χ² p-value	χ²	OR	RR
Lived	162	93.6	46 (28.4%)	-	-	-	-	-	36 (22.2%)	-	-	-	-	-
Died	11	6.4	7 (63.6%)	0.0141	-	-	4.4	2.2	7 (63.6%)	0.0021	-	-	6.1	2.9
Intensive care <2 days	76	43.9	9 (11.8%)	-	-	-	-	-	5 (6.6%)	-	-	-	-	-
Intensive care ≥2 days	97	56.1	44 (45.4%)	-	<0.0001	22.5	6.2	3.8	38 (39.2%)	-	<0.0001	24.2	9.1	6
Hospital stay ≤5 days	73	42.2	12 (16.4%)	-	-	-	-	-	7 (9.6%)	-	-	-	-	-
Hospital stay >5 days	100	57.8	41 (41.0%)	-	0.0005	12	3.5	2.5	36 (36.0%)	-	<0.0001	15.8	5.3	3.8
Adverse outcome - no	57	32.9	6 (10.5%)	-	-	-	-	-	3 (5.3%)	-	-	-	-	-
Adverse outcome - yes	116	67.1	47 (40.5%)	-	<0.0001	16.2	5.8	3.8	40 (34.5%)	-	<0.0001	17.5	9.5	6.6
Discharge commands	152	87.9	40 (26.3%)	-	-	-	-	-	30 (19.7%)	-	-	-	-	-
No discharge commands	21	12.1	13 (61.9%)	-	0.0009	11	4.6	2.4	13 (61.9%)	-	<0.0001	17.6	6.6	3.1
Three-month commands	142	82.6	40 (28.2%)	-	-	-	-	-	31 (21.8%)	-	-	-	-	-
No three-month commands	30	17.4	13 (43.3%)	-	0.1022	2.7	2	1.5	12 (40.0%)	-	0.0368	4.4	2.4	1.8

Table 4 displays the multivariate logistic regression stepwise p-values for each of the six undesirable outcome dependent variables when considering ICH ME + ICH AIS ≥5 scores and total ICH ≥6 scores as potential independent variables. All six undesirable outcomes showed a significant independent association (p < 0.0500) with total ICH scores ≥6. All six undesirable outcomes demonstrated an insignificant independent association (p > 0.1000) with ICH ME + ICH AIS scores ≥5.

**Table 4:** Stepwise multivariate logistic independent associations of ICH AIS + ICH ME score ≥5 and total ICH score ≥6 with undesirable outcomes ICH macrobleed = ICH AIS + ICH ME; total ICH = ICH macrobleed + ICH microbleed AIS, Abbreviated Injury Scale; ICH, intracranial hemorrhage; ME, mass effect

Dependent outcome	ICH AIS + ICH ME score ≥5 p-value	Wald χ²	Total ICH score ≥6 p-value	Wald χ²
Hospital death	>0.1000	5.2	0.0021	7.7
Intensive care stay ≥2 days	>0.1000	0.9	<0.0001	4.1
Hospital stay >5 days	>0.1000	0	0.0002	4.7
Adverse outcome	>0.1000	0.6	0.0003	3.6
No discharge commands	>0.1000	9.7	0.0001	14.7
No three-month commands	>0.1000	2.6	0.0404	4.2

Table 5 presents a comparison of ICH AIS + ICH ME and total ICH scores for the GCS 13-15 and GCS 3-12 groups. Both scores were greater in the GCS 3-12 group than in the GCS 13-15 cohort. The total ICH score ≥6 proportion was higher in the GCS 3-12 cohort (55.8%; 24/43) than in the GCS 13-15 group (14.6%; 19/130; p < 0.0001; OR = 7.4). Multivariate logistic regression analysis showed that the GCS 3-12 group had an independent association with total ICH (p < 0.0001), but not with ICH AIS + ICH ME (p > 0.1000).

**Table 5:** Univariate associations of ICH ME, ICH AIS, and total ICH scores with the GCS score ICH macrobleed = ICH AIS + ICH ME; total ICH = ICH macrobleed + ICH microbleed AIS, Abbreviated Injury Scale; GCS, Glasgow Coma Scale; ICH, intracranial hemorrhage; ME, mass effect

GCS group	Number	ICH AIS + ICH ME score median (IQR)	Wilcoxon p-value	Total ICH score median (IQR)	Wilcoxon p-value
GCS 13-15	130	4 (2-4)	-	4 (3-5)	-
GCS 3-12	43	5 (4-6)	0.0004	6 (4-7)	<0.0001

Table 6 presents the multivariate logistic regression analysis of independent associations between total ICH scores and outcomes. Total ICH scores had an independent association with hospital mortality, increased ICU stay, increased hospital stay, an adverse outcome, and not following commands at three months.

**Table 6:** Multivariate independent associations of total ICH scores with outcomes ICH macrobleed = ICH AIS + ICH ME; total ICH = ICH macrobleed + ICH microbleed AIS, Abbreviated Injury Scale; GCS, Glasgow Coma Scale; ICH, intracranial hemorrhage; ME, mass effect

Dependent outcome	GCS 3-12 p-value	Wald χ²	AIS + ME p-value	Wald χ²	Total ICH p-value	Wald χ²	R²
Hospital death	>0.1000	1.2	>0.1000	1.1	0.0151	5.9	0.05
Intensive care stay ≥2 days	0.0004	12.5	>0.1000	0.3	0.0004	12.4	0.29
Hospital stay >5 days	0.0004	12.7	>0.1000	0	0.0071	7.2	0.18
Adverse outcome	0.0031	8.7	>0.1000	0.2	0.0046	8	0.18
No discharge commands	0.0001	23.6	>0.1000	1	>0.1000	1.8	0.21
No three-month commands	0.8283	0	0.0948	2.8	0.0443	4.1	0.03

The GCS deficit was greater in those not following commands at discharge (median 9; IQR 5-12; n=21) than in those following commands (median 0; IQR 0-1; n = 152; Wilcoxon p < 0.0001). Table 7 demonstrates that the median GCS deficit + total ICH score was markedly greater in those not following commands at discharge than in those following commands. For the 21 patients not following commands at discharge, the GCS deficit + total ICH score was higher (median 15; IQR 11-18) than the GCS deficit alone (median 9; IQR 5-12; Wilcoxon p = 0.0001).

**Table 7:** Univariate association of GCS deficit + total ICH score with discharge commands Total ICH = ICH AIS macrobleed + ICH ME macrobleed + ICH microbleed AIS, Abbreviated Injury Scale; GCS, Glasgow Coma Scale; ICH, intracranial hemorrhage

Variable	No.	%	GCS deficit + total ICH score median (IQR)	Wilcoxon p-value
Discharge commands	152	87.9	5 (3-6)	-
No discharge commands	21	12.1	15 (11-18)	<0.0001

Table 8 shows that GCS deficit + total ICH score had an independent association with not following commands at hospital discharge in multivariate logistic regression analysis.

**Table 8:** Multivariate independent association of GCS deficit + total ICH score with discharge commands ICH macrobleed = ICH AIS + ICH ME; total ICH = ICH macrobleed + ICH microbleed AIS, Abbreviated Injury Scale; GCS, Glasgow Coma Scale; ICH, intracranial hemorrhage; ME, mass effect

Dependent outcome	GCS 3-12 p-value	ICH AIS + ICH ME score p-value	GCS deficit + total ICH score p-value	r-square
No discharge commands	>0.1000	>0.1000	<0.0001	0.24

## Discussion

The study group consisted of 173 trauma center patients admitted with ICH and blunt trauma who had undergone postinjury brain MRI. This represents a small but substantial proportion of ICH patients with a hospital stay ≥3 days. Common indications for MRI in the current study included persistent coma, motor deficits (unsteady gait, focal facial weakness, and hemiparesis), pre-ICH confusion, new-onset post-ICH confusion, neurologic deterioration, and a brain mass on CT.

A recent publication provided insight into perspectives and recommendations from 81 multidisciplinary experts regarding the use of MRI in traumatic brain injury [50]. Importantly, the panel noted a lack of consensus regarding the routine use of MRI protocols for evaluating and managing patients with traumatic brain injury. Specific recommendations for obtaining MRI scans included severity of brain injury, particular clinical manifestations (e.g., headache, vomiting, seizures, amnesia, motor deficits, affective disorders, or skull fractures), and extremes of age.

WMH and WMM proportions without ICH

Results from nine published WMH proportions, according to age, in non-head-injured counterparts are presented in Table 9. WMH proportions typically ranged from 33.0% to 36.9% for ages 31-45 years and from 85.1% to 94.9% for ages ≥70 years.

**Table 9:** Published non-head-injured WHM prevalences sorted by age WHM, white matter hyperintensity

Study	Population	Age (year)	Total	Positive	%
Han et al. (2018) [9]	Excluded stroke	35-39	38	13	34.2
		40-49	299	140	46.8
		50-59	460	322	70
		55	1211	848	70
		60-69	309	263	85.1
		70-80	105	99	94.3
Mu et al. (2022) [10]	Healthy	40-45	56	36	64.3
		46-50	66	46	69.7
		51-55	99	79	79.8
		56-60	79	67	84.8
		61-65	54	46	85.2
		66-70	45	38	84.4
de Leeuw et al. (2001) [36]	Excluded dementia	60-70	464	315	67.9
		70-80	415	353	85.1
		80-90	196	186	94.9
Kynast et al. (2018) [37]	Community	60	849	484	57
Liao et al. (1996) [38]	Community	62	1920	1632	85
Rodriguez et al. (2024) [39]	Hospital patients	40-54	151	35	23.2
		55-64	167	92	55.1
		65-74	202	141	69.8
		75+	190	172	90.5
Tate et al. (2024) [40]	Post-combat	40	198	73	36.9
Wang et al. (2019) [41]	Healthy	31-45	848	280	33
Wen and Sachdev (2004) [42]	Healthy	60-64	477	477	100
Wen et al. (2009) [43]	Healthy	44-48	428	218	50.9

Results from eight published WMM proportions, according to age, in non-head-injured counterparts are presented in Table 10. WMM proportions typically ranged from 6.5% to 15.3% for ages 45-60 years and from 23.9% to 39.4% for ages ≥80 years.

**Table 10:** Published non-head-injured WMM prevalences sorted by age WMM, white matter microbleed

Study	Population	Age (year)	Total	Positive	%
Poels et al. (2010) [11]	Population-based	45-50	413	27	6.5
		50-59	1696	195	11.5
		Mean 60	3979	609	15.3
		60-69	1350	227	16.8
		70-79	377	109	28.9
		>80	143	51	35.7
Luo et al. (2021) [12]	Excluded brain disease	55-64	40	3	7.5
		64-74	102	11	10.8
		Mean 71	199	25	12.6
		75-92	57	11	19.3
Graff-Radford et al. (2019) [44]	Population-based	60-69	485	55	11.3
		≥60	1215	274	22.6
		70-79	390	85	21.8
		≥80	340	134	39.4
Jali et al. (2024) [45]	Excluded dementia	50-60	887	70	7.9
		Mean 64	2599	375	14.4
		60-70	1025	149	14.5
		70-80	556	112	20.1
		>80	131	87	33.6
Lu et al. (2021) [46]	Healthy community	Mean 62	8159	572	7
Sveinbjornsdottir et al. (2008) [47]	Stroke 9%	Mean 76	1962	218	11.1
Vernooij et al. (2008) [48]	Population-based	Mean 70	1062	189	17.8
Yubi et al. (2018) [49]	Elderly community	65-69	356	42	11.8
		70-74	335	58	17.3
		Mean 75	1281	240	18.7
		75-79	282	60	21.3
		80-84	188	45	23.9
		≥85	120	35	29.2

WMH and WMM proportion comparisons

The age of the 173 patients with ICH who underwent MRI was 64.7 ± 19.9 years (range, 19-94). The WMH proportion among patients with ICH was 67.6% (117/173; 95% CI, 60.3-74.2%). Non-head-injured literature WMH proportions for age-matched counterparts are presented in Table 11. The combined random-effects WMH proportion was higher in the non-head-injured group (83.1%) compared with the ICH group (67.6%; Fisher’s exact p < 0.0001; OR, 2.4; 95% CI, 1.7-3.3).

**Table 11:** Non-trauma WHM prevalences for ages matching the trauma cohort The 83.1% combined study positive proportion was the random-effects result. WHM, white matter hyperintensity

Study	Age (year)	Total	Positive	%	95% CI
Han et al. (2018) [9]	60-69	309	263	85.1	-
Mu et al. (2022) [10]	61-65	54	46	85.2	-
de Leeuw et al. (2001) [36]	60-70	464	315	67.9	-
Kynast et al. (2018) [37]	Mean 60	849	484	57	-
Liao et al. (1996) [38]	Mean 62	1920	1632	85	-
Wen and Sachdev (2004) [42]	60-64	477	477	100	-
Combined	-	4073	3385	83.1	66.6-94.7

Age was higher in patients with WMH (72.4 ± 14.0 years) than in those without WMH (48.6 ± 20.9 years; p < 0.0001; Cohen d = 1.3). The WMH proportion was greater in patients with GCS 13-15 (73.9%; 96/130) than in those with GCS 3-12 (48.8%; 21/43; p = 0.0024; OR = 3.0). Patients with GCS 13-15 were older (69.7 ± 16.8 years) than those with GCS 3-12 (49.5 ± 21.0 years; t-test p < 0.0001; Cohen d = 1.1). Multivariate logistic regression analysis showed that WMH had an independent association with age (p < 0.0001) but not with GCS 3-12 (p = 0.6911).

The WMM proportion among patients with ICH was 55.5% (96/173; 95% CI, 48.1-62.7%). Non-head-injured literature WMM proportions for age-matched counterparts are presented in Table 12. The combined fixed-effects WMM proportion was lower in non-head-injured individuals (14.7%) compared with the ICH group (55.5%; Fisher’s exact p < 0.0001; OR = 7.2; 95% CI, 5.3-9.9). Age was similar in patients with WMM (62.8 ± 20.8 years) and without WMM (67.1 ± 18.6 years; p = 0.1577). The WMM proportion was higher in patients with GCS 3-12 (76.7%; 33/43) than in those with GCS 13-15 (48.5%; 63/130; p = 0.0012; OR = 3.5).

**Table 12:** Non-trauma WMM prevalences for ages matching the trauma cohort The 14.7% combined study positive proportion was the fixed-effects result. WMM, white matter microbleed

Study	Age (year)	Total	Positive	%	95% CI
Poels et al. (2010) [11]	60-69	1350	227	16.8	-
Graff-Radford et al. (2019) [44]	60-69	485	55	11.3	-
Jali et al. (2024) [45]	60-70	1025	149	14.5	-
Yubi et al. (2018) [49]	65-69	356	42	11.8	-
Combined	-	3216	473	14.7	13.5-16.0

The age of the 130 patients with ICH, GCS 13-15, and MRI was 69.7 ± 16.8 years. The WMM proportion for these patients with ICH was 48.5% (63/130; 95% CI, 40.0-57.0%). Non-head-injured literature WMM proportions for age-matched counterparts are presented in Table 13. The combined fixed-effects WMM proportion was lower in non-head-injured individuals (17.1%) compared with the GCS 13-15 ICH group (48.5%; Fisher’s exact p < 0.0001; OR = 4.6; 95% CI, 3.2-6.6).

**Table 13:** Non-trauma WMM prevalences for ages matching the GCS 13-15 trauma cohort The 17.1% combined study positive proportion was the fixed-effects result. GCS, Glasgow Coma Scale; WMM, white matter microbleed

Study	Age (year)	Total	Positive	%	95% CI
Luo et al. (2021) [12]	Mean 71	199	25	12.6	-
Vernooij et al. (2008) [48]	Mean 70	1062	189	17.8	-
Yubi et al. (2018) [49]	70-74	335	58	17.3	-
Combined	-	1596	273	17.1	15.3-19.0

The age of the 43 patients with ICH, GCS 3-12, and MRI was 49.5 ± 21.0 years. The WMM proportion for these patients with ICH was 76.7% (33/43; 95% CI, 62.3-86.8%). Non-head-injured literature WMM proportions for age-matched counterparts are presented in Table 14. The combined random-effects WMM proportion was lower in non-head-injured individuals (8.8%) compared with the GCS 3-12 ICH group (76.7%; Fisher’s exact p < 0.0001; OR = 34.2; 95% CI, 16.6-70.1).

**Table 14:** Non-trauma WMM prevalences for ages matching the GCS 3-12 trauma cohort The 8.8% combined study positive proportion was the random-effects result. GCS, Glasgow Coma Scale; WMM, white matter microbleed

Study	Age (year)	Total	Positive	%	95% CI
Poels et al. (2010) [11]	45-50	413	27	6.5	-
	50-59	1696	195	11.5	-
Jali et al. (2024) [45]	50-60	887	70	7.9	-
Combined	-	2996	264	8.8	6.0-11.9

The OR was larger in patients with GCS 3-12 versus age-matched counterparts (34.2) than in patients with GCS 13-15 versus age-matched counterparts (4.6). For these two comparisons, the 95% confidence intervals for the ORs of the GCS 3-12 (16.6-70.1) and GCS 13-15 (3.2-6.6) cohorts did not overlap.

The combined literature WMH proportion for age-matched counterparts without ICH was greater than that in the ICH MRI group. Notably, the WMH proportion in the ICH MRI group was not greater than that in age-matched non-head-injured counterparts. These findings indicate that patient age, rather than head trauma, primarily accounts for the presence of WMH in the ICH MRI group. This conclusion is supported by the observation that patients with WMH were substantially older than those without WMH. If WMH were associated with head trauma, one would expect a higher prevalence in patients with lower GCS. However, the WMH proportion was greater in the GCS 13-15 cohort than in the GCS 3-12 group, suggesting that WMH was not related to head trauma. In agreement, one study on brain axonal injury explicitly stated that “WMHs were not interpreted as trauma-related and not recorded as an indication of axonal injury” [6].

The WMM proportion for patients with ICH was substantially greater than that of published age-matched counterparts without ICH. Ages were similar in patients with and without WMM. Furthermore, the WMM proportion was higher in the GCS 3-12 group than in the GCS 13-15 group. These data suggest that WMM is associated with head trauma rather than age.

Subset analyses showed that ICH patients with GCS 13-15 had a WMM proportion higher than published age-matched counterparts without ICH. The same pattern was observed for ICH patients with GCS 3-12. The OR was much larger for GCS 3-12 patients compared with their age-matched counterparts than for GCS 13-15 patients compared with theirs. The 95% confidence intervals for the ORs of the GCS 3-12 and GCS 13-15 comparisons did not overlap. These data indicate that WMM presence was increased in both GCS cohorts, with a substantially greater increment in the GCS 3-12 group, providing additional evidence that WMM is associated with head trauma.

ICH patient associations with outcomes

ROC curve analyses were used to determine cut points for undesirable outcomes: ICH ME + ICH AIS ≥5 and total ICH ≥6. For the six undesirable outcomes, such as hospital mortality, ICU stay ≥2 days, hospital stay >5 days, adverse outcome, no commands at hospital discharge, and no commands at three months, ICH ME + ICH AIS ≥5 showed univariate associations with five outcomes, whereas total ICH ≥6 was associated with all six outcomes. Measures of association (OR and RR) were higher for total ICH ≥6 than for ICH ME + ICH AIS ≥5 across all six outcomes. Stepwise logistic regression analyses selected total ICH ≥6 as more statistically relevant than ICH ME + ICH AIS ≥5 for all six outcomes.

The ICH ME + ICH AIS score reflects macrobleed traits visible on CT, while the total ICH score includes these macrobleed traits plus microbleed characteristics detected on MRI. These findings indicate that including microbleed information in addition to macrobleed status improves the statistical associations with undesirable outcomes. Therefore, the presence of WMM (axonal injury) should be considered an important risk factor in patients with CT-detected ICH.

Although ICH AIS and ICH ME scores were significantly associated, the correlation was only moderate, suggesting that these are related but functionally distinct variables. Significant associations of GCS 3-12 with both ICH ME + ICH AIS and total ICH indicate that these scores are clinically relevant. The independent association of GCS 3-12 with total ICH, but not with ICH AIS + ICH ME, provides further evidence that simultaneous documentation of macrobleed and microbleed ICH is functionally important.

In prior literature, the typical post-traumatic brain axonal injury outcome is reported using the six-month GOS Extended [2,3,5,7,32]. Admission GCS has been associated with six-month outcomes in four of five studies [2,3,7,32]. The current study adds several additional undesirable outcomes compared with previous reports.

In the current study, associations between undesirable outcomes and CT-detected macrobleed magnitude (AIS + ME) are consistent with prior investigations. Multiple studies have shown that higher head AIS scores are associated with increased hospital mortality in trauma patients [13-15]. Additional research has demonstrated that elevated head AIS scores correlate with longer intensive care stays [14] and unfavorable post-discharge outcomes [16]. ME, as an element of CT scoring, has also been linked to in-hospital mortality [17-21] and unfavorable post-hospital outcomes [21-25]. Moreover, in the present study, ME scores were associated with the need for emergency surgical decompression [26].

A review of AIS coding for the study patients revealed that over 90% of the highest head AIS codes were based on CT characteristics of intracranial macrohemorrhage. Each ICH type (e.g., subdural hematoma, cerebral contusion) has a family of relevant AIS codes, from which the coder selects a specific AIS code and its associated score based on the reported magnitude of the ICH (tiny, small, moderate, large, or massive). Thus, nearly all AIS scores were derived from categorical descriptions of ICH size on CT.

The magnitude of ICH is a primary element when assigning head AIS values [51-54]; however, fractures and/or altered levels of consciousness may also influence scoring. To examine this in the current cohort, the frequency with which ICH macrobleed on CT was used to assign the head AIS score was assessed.

A multivariate logistic regression analysis evaluated independent associations of GCS 3-12, AIS + ME, and total ICH with six undesirable outcomes. Total ICH demonstrated independent associations with five outcomes, GCS 3-12 with four outcomes, and AIS + ME with none. For the five outcomes where total ICH had an independent association, GCS 3-12 also showed independent associations for three outcomes but not for two. These findings reinforce the clinical relevance of total ICH as a risk factor.

The GCS is widely recognized for predicting undesirable outcomes in traumatic brain injury. Early post-injury GCS has been associated with in-hospital mortality [22,27,28], and lower post-injury GCS has been linked to unfavorable post-discharge outcomes [22-24,27-30].

To further explore predictors of not following commands at discharge, an interaction between GCS and total ICH was analyzed. The GCS deficit score (15 minus the GCS score) was summed with the total ICH score. Patients who did not follow commands at discharge had a markedly higher median GCS deficit + total ICH score than those who did follow commands. Multivariate logistic regression showed that failure to follow commands at discharge was independently associated with the GCS deficit + total ICH score, but not with GCS 3-12 alone or AIS + ME. This interaction indicates that brain function, macrobleed status (AIS + ME), and microbleed presence (WMM) together influence the ability to follow commands at hospital discharge. A similar observation has been reported previously for the combined effect of GCS deficit and CT macrobleed score on discharge command-following [26].

Limitations of the study

The main limitation of this study is its retrospective design. We did not provide a detailed itemization of neurologic traits for patients who did or did not undergo MRI. Additionally, the timing of the MRI relative to the ICH event (acute, subacute, or chronic phase) was not specified. A more comprehensive, systematic literature review might identify additional publications on age-associated WMH and WMM proportions, potentially altering the reference values used in this study. To assess microbleeds, some patients underwent susceptibility-weighted imaging (SWI) and others gradient echo sequences. This heterogeneity could have influenced the observed proportion of microbleeds. If all assessments had used SWI exclusively, the reported microbleed prevalence might differ. The findings are most applicable to blunt trauma ICH patients selected to undergo MRI. Because of the retrospective nature of the study, causality cannot be definitively established. Additionally, comorbidities that may have influenced patient outcomes were not considered.

## Conclusions

This study indicates that WMMs are largely sequelae of head trauma (post-traumatic brain axonal injury), whereas WMHs are primarily associated with patient age. The combination of CT-detected macrobleeds (AIS + ME) and MRI-detected microbleeds (WMM) demonstrated independent associations with poor outcomes. In contrast, macrobleeds detected by CT alone, without microbleed information, were not independently associated with outcomes. Future research should validate these findings and compare detailed neurologic characteristics between patients who do and do not undergo MRI following traumatic brain injury. Trauma center leaders should consider developing guidelines that clearly define the indications for MRI in patients with head injuries.
